# Epidemiology of Kidney Stones

**DOI:** 10.3390/healthcare11030424

**Published:** 2023-02-02

**Authors:** Kyriaki Stamatelou, David S. Goldfarb

**Affiliations:** 1 “MESOGEIOS” Nephrology Center, Haidari and Nephros.eu Private Clinic, 11527 Athens, Greece; 2 Nephrology Division, NYU Langone Health and NYU Grossman School of Medicine, NY Nephrology Section, NY Harbor VA Healthcare System, New York, NY 10016, USA

**Keywords:** calculi, renal, citrate, calcium, nephrolithiasis, oxalate, urolithiasis

## Abstract

In the past two decades, major breakthroughs that improve our understanding of the pathophysiology and therapy of kidney stones (KS) have been lacking. The disease continues to be challenging for patients, physicians, and healthcare systems alike. In this context, epidemiological studies are striving to elucidate the worldwide changes in the patterns and the burden of the disease and identify modifiable risk factors that contribute to the development of kidney stones. Our expanding knowledge of the epidemiology of kidney stones is of paramount importance and largely upgrades the modern management of the disease. In this paper, we review the variables affecting prevalence and incidence, including age, gender, race, ethnicity, occupation, climate, geography, systemic diseases, diabetes, vascular disease, chronic kidney disease, and dietary risk factors relevant to kidney stones.

## 1. Introduction

Kidney stones (KS) are a common urological disease entailing the formation and occasional passage of crystal agglomerates in the urinary tract. It is also called nephrolithiasis or urolithiasis from the Greek words nephros, for kidney, uro-, for urinary, and lithos, for stone. Kidney stones first appear in ancient Mesopotamian medical texts between 3200 and 1200 BC [1].

The Greek physician and author Hippocrates (460–377 BC) described the symptoms of bladder stones and, in his famous Oath of Medical Ethics for physicians, discouraged “cutting for the stone”, which, as he emphasized, should only be conducted by “specialists of the work” [2].

The epidemiology of KS presents with global variations that depend on geographic, socio-economic, and climate factors. Moreover, age, sex, race, and diet affect the prevalence and incidence of the disease. Obesity and metabolic syndrome are identified as risk factors for KS. The type of stones formed and their recurrence rate are also affected by the above parameters. Calcium oxalate continues to be the dominant component of KS globally.

KS are currently recognized as a risk factor for other systemic diseases such as diabetes, cardiovascular disease [3,4,5], bone fractures [6], and chronic kidney disease [7,8]. Vice versa, these conditions also are risk factors for kidney stones. It is likely that shared risk factors contribute both to kidney stone formation and these systemic conditions.

In the last three decades, the prevalence of KS has increased worldwide [9,10,11].

Prevalence rates differ between economically developed and developing countries, partly reflecting the more frequent detection of asymptomatic KS in the former. Increased salt and protein consumption, and the rising prevalence of metabolic syndrome, have been associated with the higher KS prevalence in developed countries, while malnutrition and water deprivation may contribute to the increase in developing countries.

The increased prevalence of KS is followed by a significantly higher financial burden for healthcare systems [12].

Swift changes in the global population and socio-economic and climate conditions are expected to transform the map of KS epidemiology in the years to come worldwide.

## 2. Prevalence

The latest epidemiological studies clearly confirm previous results showing an increasing prevalence of KS worldwide.

The National Health and Nutrition Examination Survey (NHANES) was used to determine the prevalence of KS in the US population. The NHANES is conducted periodically in a probability sample of the civilian non-institutionalized United States population to determine the health status of the population. Information on socio-demographic factors, health-related behaviors, medical history, use of medications, and food consumption were collected during standardized interviews of participants. The question in reference was, “Have you ever had kidney stones?”, clearly identifying symptomatic stone formers. In the modern NHANES, a new question was added “How many times have you passed a kidney stone?” in an effort to distinguish between a symptomatic and incidental finding of an asymptomatic stone.

In a comparison of NHANES II (1976–1980) and NHANES III (1988–1994), it was estimated that kidney stone lifetime prevalence increased from 3.8 to 5.2% for the 20 years period covered by the surveys [9].

The analysis of NHANES data from 2007 to 2010 that followed reported the weighted overall prevalence of kidney stone disease to be 8.8% [10].

An updated NHANES analysis initially demonstrated a slight decrease from 8.7% in 2007–2008 to 7.2% in 2011–2012, but in the next time periods, further increases were observed: from 9.0% in 2013–2014 to 10.1% in 2015–2016. The weighted and age-standardized prevalence of KS for the whole period 2007–2016 was 9.3% [13].

Another intermediate analysis of the years 2013–2014 NHANES cycle confirmed the overall prevalence of KS to be 10.1% [14].

In Germany, the prevalence of kidney stones, as assessed by a survey, had considerably increased from 4.0 to 4.7% between 1979 and 2001 [15]. In Europe, Spain and Italy documented increases in prevalence from 0.1 and 1.17 to 10 and 1.72, respectively [16,17]. In France, 9.8% of adults above 45 years report a history of kidney stones [18].

Buenos Aires in Argentina, Thebes in Greece, Northeast Thailand, Seoul in Korea, and the Balearic Islands in Spain reported KS one-year-prevalence rates of 3.96%, 15.2%, 16.9%, 5.0% and 14.3%, respectively [11].

In a meta-analysis of 58 studies, Liu et al. reported that in West Asia, Southeast Asia, South Asia, South Korea, and Japan, the prevalence of KS is 5–19.1%. These areas in Asia are considered to form a “stone belt. In most other parts of East and North Asia, the prevalence of KS is found to be lower, 1–8% [19]. The prevalence and incidence of KS have increased in most parts of Asia in recent decades [20,21]. In China in 2013, the adjusted prevalence rate for KS was 5.8%, with a steady rise observed after 1978 [22]. In Japan, the prevalence of KS increased from 4.3% in 1965 to 9.0% in 2005 [21]. South Korea also observed a rise in prevalence from 3.5 to 11.5% between 1998 and 2013 [23]. The prevalence of KS in the adult population of southern Iran was estimated to be 21.11% [24]. The highest prevalence in Asia was seen in Saudi Arabia 6.8–19.1%, with a rising trend from 6.8 to 19.1% in the period 1989 to 2008 [25]. Rising trends have also been documented in the United Arab Emirates, Kuwait, Iran, and Israel [19,26].

The heterogeneity of epidemiological data and the methodological variations prevent us from making direct comparisons of prevalence and incidence. Nevertheless, such information is indicative of the rising global trend of the disease.

## 3. Incidence

The incidence of KS has been calculated in specific populations retrospectively using medical records review or diagnostic codes.

The incidence of symptomatic KS in the residents of Olmsted County, MN, USA, has increased from 95 per 100,000 person-years to 254 per 100,000 person-years from 1984 to 2012 [27].

Kidney stone incidence for children in the same area showed a dramatic increase from 7.2 per 100,000 person-years to 14.5 per 100,000 person-years from 1984 to 2008 [28].

Smaller increases were observed for residents of Wisconsin [29] and residents of South Carolina [30].

In Europe, the incidence of kidney stones in the population of Iceland was assessed by medical record reviews. It increased in adults from 108 per 100,000 person-years in 1985–1989 to 138 per 100,000 person-years in 2005–2008 [31]. In Icelandic children, the incidence rose from 3.7 per 100,000 person-years in 1985–1989 to 8.7 per 100,000 person-years in 2010–2013 [32]. In Italy, the 1993 incidence of KS was reported to be 1.7/1000 inhabitants [17]. In Germany, the incidence of kidney stones, as assessed by a survey, had significantly increased from 120 per 100,000 persons-years to 720 per 100,000 person-years in the period from 1979 to 2000 [15]. In recent years, the overall annual incidence for KS in Germany appears to have remained relatively stable at 0.147% in 2005 and 0.153% in 2016 with a male:female ratio of 2:1. Increasing numbers were noted for patients greater than 80 years old [33].

In Japan, the incidence from 1965 to 2005 increased from 54.2/100,000 to 114.3/100,000 [21]. A dramatic rise in incidence has been reported in India and Malaysia, from lower than 40/100,000 inhabitants in the 1960s, to 930/100,000 and 442.7/100,000, respectively, 30 years later [34,35]. In Korea, the 11-year cumulative incidence in a large national sample from 2002 to 2013 was 5.71% [23].

In Australia, the annual incidence of kidney stone disease is estimated to affect 131 per 100,000 population [36].

Epidemiological information on KS from densely populated areas such as sub-Saharian Africa, Indonesia, and Brazil is very limited or lacking [20].

## 4. Age and Gender

Age, gender, and racial and ethnic differences in the prevalence of KS have been repeatedly documented.

KS prevalence increases with age. The highest prevalence, 19.7%, was found in male individuals older than 80 years, followed by 18.8% in men 60–79 years, 11.5% in men 40–59 years, and 5.1% in men 20–39 years of age [13].

Men have a higher KS prevalence in the NHANES data from 2007 to 2010, 10.6% among men, compared with 7.1% among women [37]. Another NHANES study reports a 13.0% prevalence in men and a 9.8% in women for the period 2015–2016 [13].

The KS gender gap between men and women appears to be narrowing [38]. Male prevalence is still higher but rather stable, while female prevalence shows a continuous increase through all studies and year cycles. This trend was confirmed in the latest 2017–2018 NHANES cycle, where the prevalence of KS was stable among men of all ages as well as in each age group. In contrast, the prevalence of KS among women increased from 6.5 to 9.4% for the total female population and specifically for women younger than 60 years of age. Kidney stone prevalence did not increase for women over 60 years of age [39]. There is only one study showing a higher prevalence in women 20–39 years old in the last 2013–2014 NHANES cycle; 7.5% in women compared to 4.5% in men [14]. The prevalence in men and women older than 60 years old is relatively stable over time in all studies [14].

## 5. Pediatric Population

The overwhelming rise in the occurrence of nephrolithiasis among children and adolescents in the United States in the past 30 years has been widely documented [16,40,41]. The higher rate of increase over time was seen in adolescents, pre-adolescents, and white children [30,42]. In a study looking at South Carolina, the incidence of nephrolithiasis among children aged 0 to 18 years presenting to emergency departments increased from 7.9 per 100,000 children in 1996 to 18.5 per 100,000 children in 2007 [43]. In general, the majority of healthcare utilization due to kidney stones in the pediatric population was in children aged 15–17 years [44].

In contrast to adults, female adolescents have the highest KS rates in all studies consistently. Girls were significantly more likely than boys to be hospitalized for kidney stone disease, indicating a higher incidence of symptomatic stones [45].

In a recent study, the southern region of the United States was found to be the most common geographic region for all symptomatic pediatric stone occurrences, commonly referred to as the stone “belt” [46]. Children follow adult nephrolithiasis patterns regarding race and kidney stone composition. Nephrolithiasis is higher in non-Hispanic white children than in children of African American or Hispanic descent [41].

In contrast with adults, obesity does not seem to be as clear a risk factor for stones in children [47].

In children, calcium oxalate constitutes the main component in 73% of stones. Calcium phosphate is the main component in only 9% and struvite in 13%. Uric acid is identified in 49% of stones but is not the main component in any of them [48].

The rising incidence of nephrolithiasis in children poses significant challenges for the future of healthcare systems, not only in terms of resources and costs but also in terms of a higher burden of co-morbidities that relate to nephrolithiasis.

## 6. Race and Ethnicity

In this section, we examine the role of race and ethnicity and their association with kidney stone disease. We acknowledge that race is a social construct and a poor proxy for genetic diversity. In fact, the differences in KS prevalence among these racial and ethnic categories should in no way be taken as evidence of genetic differences. More likely, they represent differences in a variety of cultural and socioeconomic variables. Data on the prevalence of kidney stones across racial groups are only available for the US, where current race descriptions are as follows: non-Hispanic white, non-Hispanic Black, Hispanic, and others.

In older studies, looking at 2007 to 2016, non-Hispanic whites had the highest prevalence of KS, at 9.8% in 2007–2008 and increasing to 12.1% in 2015–2016; non-Hispanic Asians and non-Hispanic Blacks had the lowest prevalence at 4.4–4.6% and 4.8–5.7%, respectively, while Hispanics showed a slight increase from 7.6 to 9.1% for the same period [13].

One analysis of NHANES data shows increases in time for Black people [37], rising at a rate faster than in other racial/ethnic groups. However, in the latest NHANES study, the lowest prevalence was observed among non-Hispanic Blacks and Hispanics, while no significant time trends were observed for the prevalence of KS across any race [39].

The utilization of healthcare facilities influences the detection of asymptomatic stones, consequently affecting KS reporting. It is possible that racial disparities in the prevalence of kidney stones reflect different levels of access to health care among various racial groups, therefore, affecting the accuracy of epidemiological surveys.

## 7. Occupation

Evidence on the occupational risk factors for nephrolithiasis is limited. Although several associations of nephrolithiasis with occupational groups have been described, no systematic reviews are available [49].

The two major categories of occupational groups at risk involve workers in hot climates causing “dehydration” and workers exposed to renal toxins. Brazilian steel mill “hot workers”, working in temperatures higher than 50 °C [50], Italian glass factory machinists [51], and outdoor workers in Singapore [52] have been found in older studies to have a considerably higher rate of kidney stones than local referent populations without heat exposure occupations. Exposure to chemicals such as cadmium [53], trimethyltin [54], oxalic acid [55], and ethylene glycole ethers [56] has been shown to predispose to KS.

## 8. Climate and Geography

Climate is undeniably involved in the development of kidney stones. From the pathophysiological point of view, the mechanisms behind this involvement are not clear, nor is the role of the particular climate elements, such as sunlight, temperature, or humidity. It is widely documented that kidney stones occur more frequently in relation to higher temperatures, i.e., hot areas, warm climates, or summer months [57,58].

This is probably due to a higher volume of trans-dermal insensible losses of water. When limited water intake happens, especially where drinking water is not freely available, the result is concentrated urine, a possible supersaturation of calcium, oxalate, uric acid, and phosphate and the promotion of urinary crystallization [59].

The Southeastern states of the US are described as a “stone belt” based on the higher risk for kidney stones among their residents, as previously documented [60]. This “stone belt”, although its existence was not confirmed in all temporal studies [9], has served as a major argument in favor of the influence of climate and heat on kidney stone presentation.

In his literature review, Fakheri confirms that heat plays a role in KS pathogenesis in certain populations and that this role is much greater in men than in women [61]. A study of four major metropolitan areas (Atlanta, Chicago, Dallas, and Philadelphia) showed that increased episodes of symptomatic kidney stones were associated with a temperature of 30 °C as opposed to lower numbers of episodes associated with a temperature of 10 °C [62].

Sex differences have been shown to exist in the association between heat and the risk of kidney stone presentation in men and women. Men had a significantly greater risk of kidney stone presentation after experiencing high temperatures than women. The authors support that this risk difference indicates a modified cumulative exposure–response relationship between daily maximum temperatures and kidney stone presentations range [63]. It is not clear whether these results are due to behavioral differences among the genders or differences in their physiological responses to higher temperatures.

Increasing greenhouse gas emissions and pollution in general, are estimated to cause an increase in average global temperatures of 1–4.5 °C [64]. Global warming and continuous climate change is expected to affect certain temperature-dependent health conditions, such as nephrolithiasis. Based on a climate model of intermediate severity warming, a prediction study has indicated a climate-related increase of 1.6–2.2 million lifetime cases of nephrolithiasis by 2050, representing an up to 30% increase in warmer US areas [65].

In a recent projection of kidney stone presentation in South Carolina under two different scenarios of climate change, one mild and one severe, a total increase of 5938 emergent kidney stone presentations attributed to heat was calculated for the period 2025 to 2089 in the mild scenario compared to a total projected increase of 10,431 emergent kidney stone presentations attributed to heat in the severe one [66]. As global ambient temperatures increase due to climate change, it is expected that the prevalence of kidney stones will increase worldwide, leading to dramatic effects on costs and economies.

The role of humidity in promoting stone formation is unsettled. As ambient temperature increases, transdermal water losses via sweat are suppressed, which could inhibit kidney stone formation. The few available data, however, suggest that there is a more complex relationship. One method, among several, of estimating temperature combined with humidity is via the wet-bulb temperature [67].

In the South Carolina database, wet-bulb temperature predicted kidney stone presentations with greater accuracy than dry-bulb temperatures in summer, suggesting that higher humidity was associated with increased kidney stone disease. It is also possible that under hot and humid conditions, the effect of high temperatures on evaporative water loss overcomes the mitigating effects of high relative humidity, resulting in increased kidney stone presentations. Perhaps this finding explains why the American southwest, with a relatively dryer but hot climate, does not seem to have the same high rates of stone prevalence affecting the American south.

## 9. KS and Systemic Diseases

Nephrolithiasis is no longer considered to be a mere symptom of a benign nature or as solely a urinary disorder. It is deemed to be a multifactorial disease linked in several ways to other systemic diseases [68,69]. Epidemiological studies have revealed the association of nephrolithiasis with systemic diseases such as obesity, metabolic syndrome [70,71,72,73,74,75,76], diabetes [72,77,78], hypertension, cardiovascular disease [4,5,79,80], and chronic kidney disease (CKD).

An analysis of the NHANES III 1988–1994 population showed that as the number of metabolic syndrome traits increased (hypertension, hypertriglyceridemia, low high-density lipoprotein, abdominal obesity, and elevated fasting glucose), the frequency of self-reported kidney stone disease generally increased from 3% when 0 traits were present to 7.5% when three traits were present and to 9.8% when five traits were present [81]. Obesity, another metabolic syndrome trait, has been associated with impaired carbohydrate tolerance, inappropriate calcium response to glucose ingestion, the increased excretion of calcium, sodium, oxalate, and uric acid, hypocitraturia, and defects in renal ammoniagenesis, all contributing to kidney stone formation [82,83]. All relevant epidemiological studies concur that obesity and a high body mass index are independent risk factors for kidney stones.

Looking at the 2007–2010 NHANES data, Scales et al. found that the prevalence of kidney stones was higher among obese, 11.2% [95% CI, 10.0–12.3], and overweight individuals, 9.2% [95% CI, 7.9–10.5], than among individuals of normal weight, 6.1% [95% CI, 4.8–7.2]. Among obese males, the prevalence of stone disease was 13.0% (95% CI, 11.0–15.1), and among obese females, the prevalence was 9.6% (95% CI, 8.3–10.8) [10].

Combining the HPFS, NHS I, and NHS II studies, Taylor et al. [70] reported a link between the increase in incidental stones and the waist circumference, BMI, and weight gain of participants. The relative risk for stone formation in men weighing more than 220 lb (100.0 kg) compared to men weighing less than 150 lb (68.2 kg) was 1.44. In older and younger women, the relative risks were 1.89 and 1.92 kg/m^2^, respectively. In men, weight gain of more than 35 lb (15.9 kg) since the age of 21 years was 1.39; in older women and in younger women, weight gain since the age of 18 was 1.70 and 1.82, respectively, all compared to those with weight gain less than 35 lb.

When compared to a BMI range of 21 to 22.9 kg/m^2^, participants with a BMI equal to or higher than 30 had an increased relative risk for kidney stone formation; for men, the relative risk was 1.33; for older women, it was 1.90; and for younger women, it was 2.09. Waist circumference was also positively associated with kidney stone risk in all population groups studied [70]. A large longitudinal cohort of 25,268 participants in Taiwan had similar confirmed findings. In the US population, the risk of developing kidney stones was also higher in participants with a higher BMI and larger waist circumferences [84].

While weight gain is consistently associated with increased rates for KS, there are no studies confirming what is intuitively considered true, that weight loss is associated with a reduced risk of KS. One such opportunity is studies looking at bariatric surgery patients. In 6 years of follow-up in Olmsted County, new stone formation significantly increased in bariatric surgery patients (11.0%) compared to the controls (4.3%). While bariatric procedures are more effective for weight loss, the introduction of other variables, particularly hyperoxaluria, possibly override the influence of lower BMI [85]. Weight loss due to caloric restriction and not surgical options has not been examined.

## 10. Diabetes

The association of diabetes with KS was demonstrated in a cross-sectional study of three large prospective cohorts, the Nurses’ Health Study I and II and the Health Professionals Follow-up Study [72]. The multivariate relative risk of prevalent stone disease in individuals with type 2 diabetes compared to individuals without diabetes was 1.38 (95% CI 1.06–1.79) in older women, 1.67 (95% CI 1.28–2.20) in younger women, and 1.31 (95% CI 1.11–1.54) in men at baseline. Prospectively, the multivariate relative risk of the incident kidney stone formation in participants with type 2 diabetes compared to participants without diabetes was 1.29 (95% CI 1.05–1.58) in older women, 1.60 (95% CI 1.16–2.21) in younger women, and 0.81 (95% CI 0.59–1.09) in men.

In the same study, a history of kidney stones was associated with an increased risk of the incidence of type 2 diabetes in men and women [72].

Analysis of the data from the Rochester Epidemiology Project showed a high prevalence of diagnosed DM, obesity, and hypertension among the nephrolithiasis cohort and a significantly increased odds ratio for each condition when compared with the controls. Among those cases with uric acid stones, 40% had diabetes, while among cases with all other types of stones, only 9% had diabetes [77].

The pathophysiological explanation for this bidirectional relationship between nephrolithiasis and diabetes most probably lies in insulin resistance that occasionally precedes the clinical presentation of diabetes and, later on, urinary disorders that are inherent to diabetes. Stone formers with diabetes have been shown to have a significantly lower urine pH and excrete significantly larger amounts of urinary oxalate compared to non-diabetic stone formers, both conditions increasing their risk for CaOx stones [86]. Moreover, acidic urine increases the specific risk for uric acid stones, the type of stones predominantly encountered in diabetic patients [87,88].

Additionally, the severity level of the disease appears to modify the risk for nephrolithiasis according to a study showing that among persons with type 2 diabetes, a more serious illness, is associated with a higher risk of kidney stones [89]. In a retrospective study, a fasting glucose level of ≥100 mg/dl was positively related to increases in renal stone size over time, possibly pointing to a link between effective diabetes management and lower KS recurrence [90].

Recent data suggest that “fatty liver” disease and nonalcoholic steatohepatitis (NASH) are associated with the hepatic down-regulation of AGXT, an enzyme whose activity reduces the generation of oxalate. Mutations of this enzyme are responsible for primary hyperoxaluria type I. The result of this enzymatic down-regulation is also the increased generation of oxalate. This effect could be responsible for the apparent increased rates of calcium oxalate stones in patients with DM, overweight, and metabolic syndrome [91].

Nephrolithiasis has also been associated with gout, possibly reflecting shared risk factors with metabolic syndrome. In a cohort of 51,529 male healthcare professionals in the US, a history of gout independently increased the risk for incident kidney stones in men (RR 2.12; 95% CI 1.22 to 3.68), while a history of kidney stones was not associated with increased risk of gout (RR 1.05; 95% CI 0.54 to 2.07) [92,93]. Because these studies lack data regarding stone composition, there is some uncertainty about whether gout increases rates of uric acid or calcium oxalate stones or both. Most patients with gout, after all, have the disorder as the result of the underexcretion of uric acid rather than overproduction.

Modern lifestyles and dietary habits are causing a higher occurrence of hyperuricemia. While hyperuricemia is considered a risk factor for gout, the role of isolated hyperuricemia in the development of kidney stones is not explored. Limited observational studies report an association between hyperuricemia and uric acid stones [94]. In a cohort study of 239,331 Korean adults who were followed up for 12 years and who were free from gout and KS, hyperuricemia was associated with increased risk for the development of KS in a dose-dependent manner (*p* for trend < 0.001) in men but not in women [95]. A recent analysis of the UK Biobank, using Mendelian randomization (MR) to reduce confounding variables, evaluated if urolithiasis represents a causal effect of hyperuricemia [96]. The study included patients without gout or prior KS and did not observe any causal effect of high serum urate levels on the incidence of urolithiasis both in unadjusted (OR 0.93, 95% CI 0.81–1.08) and adjusted (OR 0.94, 95% CI 0.80–1.09) models [96].

## 11. Vascular Disease

The risk of cardiovascular disease has been associated with a history of KS, but the pathogenic mechanisms behind such a relationship are not clear. Following older hypotheses but inconclusive studies [97,98,99], modern observations explored and confirmed an increased prevalence and incidence of hypertension in stone formers [100,101]. A higher incidence of nephrolithiasis in hypertensive patients was also observed but not consistently in all studies.

In a cross-sectional analysis of European men [102] and in the following prospective studies, Strazzullo and Cappuccio report that hypertensive patients were at higher risk of forming stones, with a relative risk (RR) of 1.89 and a 95% CI of 1.12–3.18, [103,104] In a prospective study, Borghi et al. confirmed that hypertensive patients had a significantly increased incidence of stone episodes (unadjusted OR = 5.5, 95% CI = 1.82–16.66) [105].

In their two large prospective studies, Madore et al. [100,101] were only able to confirm one arm of the association, that patients with a history of stones had a higher tendency to develop hypertension. Hypertensive patients in his studies did not have a higher incidence of kidney stones. Undeniably, nephrolithiasis is considered to be a risk factor for the development of hypertension, but little evidence is offered for the opposite stipulation: hypertension as a risk factor for the incidence of kidney stones. Perhaps KS themselves predispose to hypertension, or more likely, the physiologic mechanisms that contribute to hypertension later in life may also favor the earlier formation of kidney stones.

Looking at further aspects of cardiovascular disease (CVD), such as myocardial infarction (MI) and stroke, a study of the Portuguese population identified significant associations between KS and MI and stroke [79]. After 9 years of follow-up of residents in Olmsted County, Minnesota, stone formers had a 38% (95% confidence interval 7 to 77%) increased risk for myocardial infarction (MI), which persisted at 31% (95% confidence interval of 2% to 69%) after adjustment for CKD and other comorbidities. The authors concluded that the fact that kidney stone formation is an independent risk factor for MI implies a common pathophysiologic mechanism [5].

“CARDIA”, a US population-based observational study of 5115 white and African American men and women, observed a significant association between a history of kidney stones and subclinical carotid atherosclerosis in young adults, adding further support to the notion that kidney stones and atherosclerosis have shared pathogenic mechanisms and risk factors [106].

Ferraro et al. prospectively studied 45,748 men and 196,357 women who were participants in the Health Professionals Follow-Up Study, HPFS and the Nurses’ Health Study (NHS) I and II in the US. They found that among women, those with a reported history of kidney stones compared with those without, had an increased risk of CHD in NHS I (incidence rate (IR): 754 vs. 514/100,000 person-years also in NHS II: IR 144 vs. 55/100,000 person-years. No significant association was observed in men: IR 1355 vs. 1022/100,000 person-years [4].

In accordance with previous studies, stone formers in the Canadian healthcare system were found to have a 63% (95% CI: 1.51–1.76) higher risk of the incidence of myocardial infarction, with a greater effect in women [80].

In a prospective 5-year follow-up in Taiwan, nephrolithiasis was a significant predictor of stroke, with the stone formers being 1.43 times more likely to have a stroke than the comparison group [107]. In a meta-analysis of available studies, Liu et al. confirmed a significant association of stroke risk for kidney stone formers compared with the controls (HR, 1.40; 95% CI, 1.20–1.64) [108].

Intuitively, the link between nephrolithiasis and CVD is related to calcium metabolism disorders, but the pathophysiological pathways are not clear. Arterial calcification could be the common mechanism linking atherosclerosis with nephrolithiasis. The inappropriate osteogenesis of renal epithelial cells, similar to that of vascular cells in vascular calcification, may be promoted by urinary disorders and result in the formation of CaP crystals and Randall’s plaques [109].

In a retrospective, matched case–control study of 57 KS formers and 54 healthy controls at the Royal Free Hospital (London, UK), abdominal aortic calcification (AAC) was assessed using computed tomography (CT) imaging. The AAC severity score (median [25th percentile, 75th percentile]) was significantly higher in KS patients compared with the control group (0 [0, 43] versus 0 [0, 10], *p* < 0.001) and a multivariate model adjusted for age, sex, high BP, diabetes, smoking status, and eGFR confirmed that KS formers had higher AAC scores compared with non-stone formers (*p* < 0.001) [110].

At the molecular level, the dysfunction of the calcium-sensing receptor (CaSR) might be the missing link between nephrolithiasis and CVD. CaSR is a protein-coupled receptor widely expressed in the kidneys and the vascular system that regulates the renal handling of calcium and the signaling of calcium in the vascular system [111,112]. In another proposed mechanism, oxidative stress is the common trigger for endothelial dysfunction and chronic inflammation both in nephrolithiasis and vascular disease [113].

## 12. CKD

Nephrolithiasis is currently a noteworthy primary cause of chronic kidney disease and end-stage kidney disease (ESKD) in global renal registries. As the primary cause of ESKD is without doubt multifactorial, and CKD is confounded by several comorbidities, it has been problematic for researchers to address and exhibit these relationships methodologically and accurately. Acknowledging certain limitations, most studies have confirmed that symptomatic stone formers are at increased risk of CKD [7] and the progression of CKD to ESKD [8].

Stone formers in Olmsted County Minnesota who were diagnosed between 1986 and 2003 were followed up for a mean of 8.6 years and were found to be at increased risk for a clinical diagnosis of CKD, as defined either by clinical criteria or by a higher-than-normal serum creatinine concentration or by a lower-than-normal estimated glomerular filtration rate (eGFR). Stone formers were also found to be at increased risk for ESKD or death with CKD, but this finding was not significant [7].

The multivariate analysis of 5971 NHANES 2007–2010 database participants, of whom 521 were stone formers, demonstrated a positive association of kidney stones with CKD and CKD progression to dialysis, OR 1.50 and 2.37, respectively. Researchers defined CKD as an eGFR lower than 60 mL/min/1.73 m^2^ and/or a urinary albumin-to-creatinine ratio larger than 30 mg/gm. The association was significant in women but not in men [114].

A later study of stone formers in Olmsted County, Minnesota, looking at the years 1984 to 2012, revealed that the risk of ESKD was higher (HR, 2.34; 95% CI, 1.08–5.077) in recurrent symptomatic stone formers but not in the incidence of symptomatic kidney or bladder stone formers, suggesting a relationship between adverse outcomes and the frequency–recurrence of stone events and the resulting kidney injury [115].

Variable pathophysiologic processes and putative mechanisms via which nephrolithiasis cause chronic kidney damage and CKD have been proposed. These mechanisms differ according to the type of stones: their size and composition [116]. The glomerulosclerosis, tubular atrophy and interstitial fibrosis that are encountered during the kidney biopsies of stone patients indicate the involvement of a broad spectrum of pathophysiological processes [117].

Apart from the obvious detrimental effect of complete unilateral obstruction on renal function, transient obstruction or recurrent episodes of obstruction are deemed to cause damage to the functional nephrons, forcing the remaining nephrons to hyperfilter, consequently causing CKD [106,118].

Patients with calcium phosphate, infection-related (struvite), and uric acid stones are at the highest risk for CKD, likewise for patients with a large number or larger-size stones or staghorn calculi. It is unclear if this is due to the deposition of minerals, such as plugs at the ducts of Bellini in brushite stone formers, due to the inherent nature of the stones, or due to the magnitude of their complications [119].

Patients with nephrolithiasis of a hereditary cause, such as cystinuria, primary hyperoxaluria, renal tubular acidosis, or intestinal hypercalciuria, have a higher risk of CKD and progression to ESKD [120,121,122]. Along with frequent urological interventions, multiple shock-wave lithotripsies (SWL) sessions were initially considered to place patients at risk for a loss of renal function [123]. However, subsequent studies suggested that SWL does not directly affect the glomerular filtration rate (GFR) [124].

In a population-based retrospective study of the Health Improvement Network, urolithiasis was associated with a significant hazard ratio of 1.42 for hypertension and 1.82 for CKD, but SWL was not associated with the incidence of CKD [125]. In conclusion, it is impossible to weigh the risk of kidney damage attributed to any intervention per se as opposed to that caused by the lack of intervention.

KS cause kidney injury via multiple mechanisms, including urine obstruction and inflammation. Another proposed mechanism is crystallopathy, involving the direct toxicity of calcium oxalate crystals to the tubular epithelial cells, stimulating inflammation and leading to the production of reactive oxygen species, promoting further tubular injury [126].

Regarding the postulated mechanisms, the previously explored link between kidney stones and metabolic syndrome and diabetes might offer another explanation for the increased risk for CKD among stone formers. Metabolic syndrome might be a common causal factor for both CKD and KS, predisposing stone formers to the loss of kidney function [114].

CKD and ESKD patients have multiple comorbidities, often of greater severity than kidney stones. In the presence of these comorbidities, it is impossible to accurately weigh the role and contribution of nephrolithiasis in CKD or ESKD. In renal registries, nephrolithiasis is often overlooked as a primary cause of CKD or ESKD, thus causing an underestimation of the magnitude and the detrimental effects of the disease. The preservation of kidney function should be a major goal of all healthcare systems in order to reduce morbidity and mortality. All of the available data corroborate that the prevention and therapy of KS is a necessary step in achieving this target.

## 13. Stone Composition and Recurrence

Calcium-containing stones continue to comprise the most common KS composition globally [127]. In the population analysis of Olmsted County, Minnesota, 94% of the incidence stones were calcium stones, of which 76% were mainly calcium oxalate, 18% were mainly calcium phosphate, 5% were uric acid, 1% were struvite–magnesium ammonium phosphate, and 0.1% were cystine [128]. In Germany, the analysis of 45,783 urinary stones in the period from 2007 to 2020 produced similar results. Calcium oxalate (CaOx) was the most frequent type of stone, with 71.4%, calcium phosphate was 10.2%, and uric acid was 8.3% [129].

Uric acid nephrolithiasis accounts for 8–10% and is increasing globally. It mostly presents in obese cases or individuals with metabolic syndrome, and its increasing incidence corresponds to the increasing prevalence of metabolic syndrome, obesity, and diabetes worldwide. Uric acid stone prevalence is higher in older ages. Hyperuricosuria is not the essential causality: acidic urine is [130].

In a retrospective analysis of 1516 patients in a large stone center in the USA, the percentage of uric acid stones relative to the total number of kidney stones increased significantly from 7% to 14% over the period 1980–2015. In this study, uric acid stone formers were older and had a higher BMI and a lower urinary pH than calcium stone formers [131]. In another US study with 4339 kidney stones covering patients from seven states, uric acid stones comprised 12%. This study also showed that, with the exception of Florida, the stone composition did not differ across US regions [132]. An increase in uric-acid-containing stones from 2.0% 40 years ago to 9.1% in the 2014–2017 period was also reported in a Norwegian surgical cohort [133,134].

Struvite stones containing magnesium ammonium phosphate, also known as infection-related stones, comprise 7–8% of stones worldwide. They are the result of the ammoniagenesis caused by urea-splitting bacteria secondary to infection [135].

Rare inherited metabolic disorders are often linked to the presentation of kidney stones in early childhood, causing a high burden of recurrence. These include cystinuria, primary hyperoxaluria, distal renal tubular acidosis (RTA), xanthinuria, Lesch–Nyhan syndrome, Dent disease, and adenine phosphoribosyltransferase (APRT) deficiency (a cause of dihydroxyadenine stones).

Cystinuria, the most common of the rare monogenic disorders, accounts for 6% to 8% of stones in children and 1% to 2% of stones in adults [121,136].

Primary hyperoxalurias, with an estimated prevalence of about 1 to 3 per million population, are critical to diagnose [137]. If undiagnosed and untreated, these disorders lead to a significant recurrence of kidney stones, particularly for primary hyperoxaluria type 1 kidney failure.

So far, the epidemiological data point to uric acid and other rare stones as being responsible for the highest rate of symptomatic recurrence, rendering knowledge of stone composition essential to disease prevention.

Stone recurrence may be defined to include both clinically evident, symptomatic stones or asymptomatic, radiographically revealed imaging changes in kidney stone burden [138]. In addition to the inconsistency in terminology and classification, the recurrence rates for nephrolithiasis have been portrayed by older studies at a grim 50% [139,140]. In a community study with a 5-year follow-up in Minnesota and Florida, 19% of the patients had clinically evidenced symptomatic recurrence and 25% self-reported recurrence. Asymptomatic radiographic recurrence was also observed; a new stone in 35%, stone growth in 24%, and stone passage in 27% of patients [141].

Based on its results, this study attempted to propose a new “rule of halves”: “half of first time symptomatic stone formers present with a baseline asymptomatic kidney stone, half of these will pass the stone over the next 5 years, and half of these will have symptoms (pain or gross hematuria) with stone passage” [141].

In a random sample of the incidence of symptomatic kidney stone formers in Olmsted County, Minnesota, the recurrence rate was 3.4 (95% CI, 3.2–3.7) per 100 person-years after the first episode, 7.1 (95% CI, 6.4–7.9) after the second episode, 12.1 (95% CI, 10.3–13.9) after the third episode, and 17.6 (95% CI, 15.1–20.0) after the fourth or higher episode (*p* < 0.001 for trend). Younger age, male sex, higher body mass index, family history of stones, pregnancy, and a history of brushite, struvite, or uric acid stones were also identified as independent risk factors for higher stone recurrence [142]. A systematic review of twenty-one studies published from 1976 to 2011 reported the five-year median recurrence rate for first-time stone formers to be 26% [143].

Another comprehensive meta-analysis that included 40 retrospective and 13 prospective studies and a total of 488,130 patients studied the risk factors for kidney stone disease recurrence [144]. Twelve risk factors for recurrence were identified: younger age (*n* = 18), higher BMI (*n* = 16), family history of kidney stones (*n* = 12), personal history of kidney stones (*n* = 11), hypertension (*n* = 5), uric acid stones (*n* = 4), white race (*n* = 3), suspected kidney stone episode before the first confirmed stone episode (*n* = 3), surgery (*n* = 3), any concurrent asymptomatic (non-obstructing) stone (*n* = 2), pelvic or lower pole kidney stone (*n* = 2), and 24-h urine test completion (*n* = 2) [144].

## 14. Risk Factors for Nephrolithiasis

The risk factors for KS include family history, systemic diseases, diet, and urinary disorders.

### 14.1. Dietary Risk Factors

Major epidemiological studies have addressed the issue of dietary risk factors, concluding with almost similar results. Current scientific evidence concludes that increased fluid, fruit, and vegetable intake has a beneficial effect on kidney stone incidence, while high salt and high meat/animal protein diets are universally accepted risk factors for the formation of kidney stones. Animal protein may be relevant as a source of protons, stimulating proximal tubular citrate reabsorption, causing lower citrate and higher calcium excretion, and relevant to uric acid stones, decreasing urine pH and increasing uricosuria. Very-low dairy-calcium consumption is also deemed unsafe.

#### 14.1.1. Fluids

Increased water intake is the universally recognized therapeutic approach for reducing the risk of kidney stones.

Since water hardness has not been correlated with the incidence risk of kidney stones in population studies, it has been suggested that the important factor that modifies patients’ risk for kidney stone formation is the quantity and not the quality of water ingested [145,146].

In a recent population study using the NHANES 2009–2012 cycles and a total of 8195 adults aged 20 years or older, water intake and hydration were associated with nephrolithiasis risk. Confirming previous findings, the study associated a daily water intake of greater than 2500 mL/d and a urine output of 2 L/d with a lower prevalence of nephrolithiasis [147].

In a study analyzing three large cohorts (the Health Professionals Follow-Up Study, Nurses’ Health Study I, and Nurses’ Health Study II) with a total of 194,095 participants and an 8-year median follow-up, the risk for incident kidney stones was higher when a higher consumption of sugar-sweetened non-cola or cola beverages was reported [148]. The consumption of at least one sugar-sweetened soda daily increased the risk of stone formation by 23–33% compared to the consumption of less than one serving weekly. Conversely, participants in the highest quintile of caffeinated coffee consumption had a significantly lower risk of kidney stones [148].

In both NHS I and HPFS, greater coffee consumption was associated with a reduced risk of the incidence of stone formation, while decaffeinated coffee demonstrated a similar effect. Therefore, the beneficial effect of coffee may not be related to its caffeine content [149,150]. Similarly, in a study in the United Kingdom, each 200-cc serving of coffee per day was associated with an 8% risk reduction in the incidence of stone formation [151]. Coffee consumption was previously considered to promote kidney stone formation, perhaps due to its high oxalate content, especially in cases of enteric or idiopathic hyperoxaluria. A recent study employing Mendelian randomization exploration definitively abolished this notion, proposing a protective role for coffee consumption and corroborating previous epidemiological data [152,153].

Fruit juices have been explored in regard to their effect on the risk of kidney stone formation. Although fruit juice might confer a protective effect via an increase in urinary citrate, the data from NHS I and HPFS report an increased risk of incident stone formation among grapefruit-juice-consuming individuals. However, a subsequent paper with a longer follow-up did not confirm that effect [149,150].

With respect to the risk of the incidence of stone formation, a pooled analysis of the NHS I, NHS II, and HPFS failed to show the benefit of apple juice, while other studies on cranberry juice and lemonade provided inconsistent results [148,154,155,156,157,158].

The study of the relationship between dietary fiber, fruit, and vegetable intake, and the risk of kidney stone formation in more than 83,000 women in the Women’s Health Initiative, showed that in women with no history of kidney stones, a higher total dietary fiber, a higher fruit intake, and a higher vegetable intake were associated with a decreased risk of the incidence of kidney stone formation [157].

#### 14.1.2. Dietary Calcium

Calcium stones historically led to a recommendation to reduce the consumption of calcium sources. However, many epidemiological studies have revealed the inverse association between dietary calcium and the risk of kidney stone formation, suggesting that calcium restriction may aggravate the risk for kidney stones and, moreover, cause bone loss.

Firstly, in 1993, the analysis of 45,619 40–75-year-old male individuals without stone disease at recruitment (the Health Professionals Follow-up Study cohort) showed that lower calcium intake was associated with a 50% higher risk of kidney stone events [158]. The inverse association between dietary calcium and the risk of kidney stone formation was similarly confirmed in a study of 1976 to 1994 NHANES data, observing a reverse association of the risk of stone disease in males in the highest quartile of calcium intake compared to those in the lowest calcium intake quartile [9].

According to the Health Professionals Follow-up Study, the above inverse association between dietary calcium and the risk of stone formation no longer occurred in men aged 60 years or older [159]. A randomized trial confirmed the above results, demonstrating a significant reduction in the risk of stone recurrence of approximately 50% after five years in the group of “normal” calcium (1200 mg/day), low-salt, and low-animal-protein diet compared to a low-calcium diet (400 mg/day) [160]. The proposed mechanisms included increased urinary oxalate excretion in patients on low-calcium diets due to the fact that calcium binds with oxalate in the intestine, reducing its absorption and subsequent urinary excretion.

Although dietary calcium from both dairy and non-dairy sources seems to have a protective effect on kidney stone events [161], supplemental calcium is associated with a higher risk of the incidence of stones, especially in older women, but not younger [159,162,163,164]. In a cohort of postmenopausal women in the Women’s Health Initiative (WHI), where the risk of calcium and vitamin D supplementation was reported, the incidence of kidney stones was higher among those receiving supplementation. While the relative risk doubled, the absolute risk was small, increasing from 1 to 2% [164].

In a recent analysis of 411 incident symptomatic kidney stone formers and 384 controls seen at the Mayo Clinic between 2009 and 2018, lower dietary calcium, lower potassium, lower caffeine, lower phytate, and lower fluid intake were all associated with higher odds of an incidence of symptomatic kidney stones [165]. Moreover, lower dietary calcium and lower potassium intake were predictive of symptomatic kidney stone recurrence [165].

#### 14.1.3. Oxalate

As the majority of stone formers have calcium oxalate stones, the role of dietary oxalate intake on urinary oxalate excretion has been repeatedly assessed in large cohort studies. Oxalate is a significant dietary target for stone prevention. Oxalate-restricted diets nevertheless seem controversial since oxalate is abundant in plants, and plant-based diets are considered generally beneficial.

In terms of physiology, roughly half of the oxalate in the urine originates from food and the other half derives from endogenous production and liver metabolism [166]. Hyperoxaluria significantly contributes to the urinary supersaturation of calcium oxalate and is a risk factor for KS. Oxalate absorption by the gut is influenced by calcium presence. Accurately measuring the ingested amount of oxalate is not deemed possible due to the large variability of oxalate content in plant-based foods and the differences in soluble vs. insoluble oxalate content.

Large cohort studies have attempted to picture the effect of oxalate-rich diets on kidney stone formation risk [167,168]. An analysis of NHS I, NHS II, and HPFS showed a 21–22% increase in the risk of the incidence of kidney stones among men and older women, with the highest quintile of oxalate intake (median 287–328 mg/day). Men with low dietary calcium intake had the highest relative risk, RR 1.46, while younger women showed no association between oxalate intake and stone risk at all [169].

The DASH diet (Dietary Approaches to Stop Hypertension) has also been proposed as an effective alternative to a low-oxalate diet in reducing calcium oxalate supersaturation, especially for hyperoxaluria patients. Urinary calcium oxalate supersaturation has been found to decrease in the DASH versus the low-oxalate group (point estimate of difference, −1.24; 95% CI, −2.80 to 0.32; *p* = 0.08) in association with an increase in magnesium and citrate excretion and urine pH in the DASH versus low-oxalate group. However, this study’s aim was not to determine if kidney stone incidence would decrease [170].

Endogenous oxalate production is known to be modified by ascorbic acid (vitamin C) intake [171]. Vitamin C ingestion has been shown to increase hyperoxaluria both in normal and kidney stone formers [172]. In the HPFS, men consuming over 1000 mg of vitamin C/day) had a 41% increase in the risk of incident stones [159], and men in Sweden who were taking vitamin C supplements had a nearly two-fold increased risk of the incidence of stone disease over 11 years of follow-up [173].

#### 14.1.4. Sodium

High sodium intake is known to increase urinary calcium excretion, and low-sodium diets are generally recommended for kidney stone patients. Large cohorts have delineated the strong association of high-sodium diets with the increased risk of nephrolithiasis.

In the NHS I, sodium intake in the highest quintile was associated with a 30% greater risk of incident stone formation than in the lowest quintile [163]. In the WHI study, the highest quintile of sodium consumption was associated with a 1.61 higher relative risk of the incidence of stones [174].

In an older study of the DASH-style diet, participants with higher DASH scores (higher intakes of calcium, potassium, magnesium, oxalate, and vitamin C and lower intakes of sodium) had a reduced kidney stone risk: 0.55 (95% CI, 0.46 to 0.65) for men, 0.58 (95% CI, 0.49 to 0.68) for older women, and 0.60 (95% CI, 0.52 to 0.70) for younger women, clearly providing evidence supporting the low sodium recommendation [175].

Practice guidelines recommend a low salt intake for stone formers. The AUA urges patients to limit sodium intake and consume less than 100 mEq (2300 mg) sodium daily [176], while the updated EAU guidelines recommend a daily sodium intake not exceeding 3–5 g [177]. Reduced urine sodium and the resulting reduction in urine calcium is also considered beneficial for maintaining bone density.

#### 14.1.5. Protein

Epidemiological evidence supports the positive association between high protein intake and kidney stone risk. In a study of three large prospective cohorts, the Health Professionals Follow-up Study (HPFS) and the Nurses’ Health Studies (NHS) I and II, it was shown that a DASH-style diet, rich in vegetables and low in animal proteins, had the lowest risk of incident kidney stones [178]. Animal protein intake increased the risk of kidney stones only in men with a body mass index of less than 25 kg/m^2^ but not in women [159].

Animal protein intake was not associated with nephrolithiasis risk in a secondary analysis of 78,293 women from the prospective WHI OS (Women’s Health Initiative Observational Study) [174]. Vegetable proteins were not associated with the risk of kidney stones, even after adjustment for age and BMI, the intake of dairy protein was inversely associated with incident kidney stone disease, and only non-dairy animal proteins seemed to be harmful [179].

There was also a 33–56% lower risk of stones for those participants in the highest quintile of potassium intake, arguing for a strong inverse association between potassium intake and the risk of stones [179]. It is possible that potassium intake is simply a surrogate for alkali intake, expected to increase urinary citrate excretion. However, potassium may have some other, currently undisclosed, independent effect.

The risk of kidney stone events was estimated to be 45% lower in individuals consuming high amounts of fruits and vegetables in addition to low-fat dairy products [175]. At present, the effect of vegetarian or vegan diets on kidney stone prevalence and incidence has not been fully explored [180]. Older studies suggested a favorable effect, a 40–60% lower kidney stone prevalence in vegetarians compared with the general population [181,182].

Finally, the study of the relationship between dietary fiber, fruit, and vegetable intake, and the risk of kidney stone formation in more than 83,000 women in the Women’s Health Initiative, showed that in women with no history of kidney stones, a higher total dietary fiber, a higher fruit intake, and a higher vegetable intake were associated with a decreased risk of incident kidney stone formation [157].

Increases in urinary citrate excretion and urinary volume are expected to overcome the effect and increase oxalate excretion. Along with previous studies, this study further affirmed the beneficial role of increased fluid intake and plant-based diets and decreased dietary sodium and animal protein intake in reducing the risk of kidney stones [174].

### 14.2. Urinary Risk Factors

The pivotal role of supersaturation (SS) in the formation mechanisms of all types of stones fully justifies the exploration of urinary composition as a risk factor for the promotion of nephrolithiasis. Moreover, KS therapy specifically directed at 24-h urine disorders is reported to be more effective than empiric therapy at preventing recurrent stone episodes [183].

Urine collections are usually performed on the patients’ self-selected diets. The parameters measured include calcium, oxalate, citrate, phosphate, urate, urine volume, pH, sodium, and potassium. Dietary protein is sometimes estimated through the calculation of protein catabolic rates. The measurement of sulfate and ammonium is also helpful in estimating animal protein intake and understanding urine pH and citrate excretion [184].

In most studies, urinary abnormalities are identified in more than 93% of KS patients [185,186]. The most prevalent metabolic abnormalities are higher calciuria, ranging from 39.4 to 54.5%, higher oxaluria, ranging from 32.4 to 34.7%, and higher uricosuria, ranging from 32.3 to 45% [183,186]. Patients with higher oxaluria are significantly more likely to have a family history of kidney stone disease (71.4% vs. 28.6%, *p* = 0.013), and patients with higher calciuria are reported to be older (54.7 vs. 47.8 years, *p* = 0.018) [185]. Higher urinary calcium is considered a major risk factor for the most common type of USD, calcium stones [187].

In an attempt to define the associations between 24 h urinary calcium excretion and demographic, dietary, and other urinary factors, Taylor EN et al. [188] conducted a cross-sectional study of 3368 individuals with and without a history of kidney stones, using the population from three cohorts: the Health Professionals Follow-up Study and the Nurses’ Health Studies I and II. The study confirmed the previously reported positive KS relation between 24 h urinary calcium and urinary sodium [189,190]. Higher urinary magnesium, lower urinary potassium, higher urinary sulfate and higher urinary volume were also associated with considerably higher levels of urinary calcium [188]. Five to 11% of calcium stone formers are found to have low urinary citrate, which increases the risk for stone formation.

In a cross-sectional study of 24-h urine excretion and the risk of kidney stone formation in 3350 men and women, of whom 2237 had a history of nephrolithiasis, higher urine calcium and oxalate significantly increased the risk of stone formation in men and women. The risk of stone formation decreased with higher citrate (*p*, trend < 0.001) and higher urine volume (*p*, trend < 0.001). Higher urinary sodium (*p*, trend < 0.001) and phosphate (*p*, trend = 0.04) were significantly associated with increased risk in men [191].

High urinary oxalate, whether idiopathic or secondary to other disorders, is one of the most prominent factors associated with higher CaOx SS and KS.

Primary hyperoxalurias (PH), on the other hand, are a group of extremely rare autosomal recessive disorders involving the hepatic overproduction of oxalate, which results in markedly increased urinary oxalate excretion, usually above 80 mg/day [192].

The prevalence of idiopathic hyperoxaluria in KS patients appears to be increasing over time [193]. In a systematic literature review, hyperoxaluria rates were found to be significantly higher in non-American patients compared with American patients (40.7% vs. 23.0%; *p* = 0.018) and hyperoxaluria rates were reported to be higher in Asian countries compared with Western countries (56.8% and 23.8%; *p* < 0.001) [180]. Urinary oxalate excretion and hyperoxaluria have been found to be lower in Black individuals than in White individuals, probably due to genetic factors [194,195].

Larger bodyweight appears to increase urinary oxalate [196], and obesity is associated with higher urinary oxalate levels in both men and women. Among stone formers, obese patients have been found to have oxalate levels about 33% higher than non-obese stone-forming patients [197].

In an analysis of three large cohorts, participants with greater BMIs were found to excrete more urinary oxalate (*p* for trend < or = 0.04), uric acid (*p* < 0.001), sodium (*p* < 0.001), and phosphate (*p* < 0.001) than participants with lower BMIs. Urinary SS of uric acid increased with BMI (*p* = 0.01). However, no relation between BMI and urinary SS of calcium oxalate was confirmed [198].

The basis for the effect of greater BMI or weight gain has not been understood exactly. That metabolic syndrome and insulin resistance lower urine pH and increase the prevalence of uric acid stones has been more clear than the effect of weight on calcium stone formation. An earlier study was prescient in suggesting that higher BMI was associated with greater urinary oxalate excretion, but the biology of such a relationship was not elucidated [196].

A provocative recent study of hepatic metabolism sheds potential light on this observation. Non-alcoholic fatty liver disease (NAFLD), leading to non-alcoholic hepatosteatosis (NASH), apparently down-regulates the activity of the hepatic enzyme alanine-glyoxylate aminotransferase (AGXT), the enzyme which, when mutated, is responsible for primary hyperoxaluria type 1 [91]. The severity of NAFLD correlates with urinary oxalate excretion. By identifying a reduced capacity of the steatotic liver to detoxify glyoxylate, leading to elevated oxalate synthesis, these studies may provide a mechanistic explanation for the increased risk of kidney stones and chronic kidney disease in NAFLD patients. Whether the widespread prevalence of this phenomenon is responsible for the repeatedly demonstrated link between overweight and kidney stones requires additional observational data. 

Low urinary citrate excretion is a common abnormality occurring in a variable of 10 to 60% of all stone formers [199]. Hypocitraturia as a sole abnormality is encountered in about 10% of calcium stone formers and as a joint abnormality in about 50% [200]. Hypocitraturia is more frequent among older stone-forming patients [201] and is not different between White, Black, or Asian individuals [202].

Hypocitraturia is a reversible risk factor mostly occurring in men with USD. Women have been found to generally have higher urinary citrate, probably due to a larger absorption of dietary organic anions, leading to a higher urinary pH [203].

It is well documented that low urinary pH and low urinary volume but not hyperuricosuria, are the necessary conditions for uric acid stone formation [204]. Increasing body mass index is associated with lower urine pH, which likely results from increasing insulin resistance [205,206]. This mechanism appears to be responsible for increasing uric acid as a component of kidney stones with increasing body mass index and with the higher prevalence of diabetes [78]. Insulin resistance leads to lower urine pH and the risk for uric acid stones by causing impaired ammoniagenesis [206]. In uric acid nephrolithiasis, hyperuricosuria is rarely the sole cause of stone formation [207].

Among calcium stone formers, only ten percent of patients have hyperuricosuria as a single disorder, while up to 40% have a combination of hyperuricosuria with other metabolic abnormalities [208]. In a recent systematic literature review of metabolic risk factors for stone formation, an increase in the prevalence of hyperuricosuria was observed (17% vs. 22%; *p* < 0.0001) [209]. Hyperuricosuria, together with hyperoxaluria, hypercalciuria, and hypocitraturia, was significantly higher in males [209].

Hyperuricosuria can be caused by rare congenital conditions [210], increased dietary purine intake, systemic diseases including gout, myeloproliferative disorders, multiple myeloma, hemoglobinopathies, thalassemia, chemotherapy, and uricosuric medications [207,211]. The postulated mechanism for higher uricosuria causing calcium KS is the promotion of calcium oxalate crystallization due to its decreased solubility in the presence of uric acid, a phenomenon called “salting out” [212].

Interestingly and counter-intuitively, hyperuricosuria was inversely associated with the incidence of kidney stone formation in the population of a large prospective observational study [191]. The association observed was significantly inverse between urinary uric acid and stone formation risk in men, marginally inverse in younger women, and none in older women. These findings significantly questioned whether uric acid was, in fact, a contributor to calcium oxalate stones [191].

In an earlier randomized trial among hyperuricosuric, normocalciuric calcium oxalate stone formers demonstrated a significant (*p* < 0.05) decline in urinary uric acid excretion and a twofold reduction in stone recurrence rates among patients treated with allopurinol. This study excluded people with higher levels of urine calcium excretion; whether urate-lowering therapy affects calcium stone incidence in patients with higher urine calcium excretion has not been tested [212].

Similarly, in a non-randomized retrospective study, another uric acid-lowering drug, febuxostat, was shown to achieve a statistically significant reduction in uricosuria and promote the dissolution of uric acid stones. However, the effect on calcium stones was not investigated [213].

An interesting observation was recently made in a randomized trial of inositol supplementation to prevent the progression of Parkinson’s disease. Observational data suggested that hyperuricemia was associated with less progression, while higher serum uric acid levels could be achieved by supplementing with the precursor inositol. There was no demonstrable effect on neurological progression. However, an adverse effect was the development of kidney stones in a number of patients; those stones that were analyzed were composed of uric acid, not calcium salts.

In conclusion, although large epidemiological studies do not corroborate the risk, at least one older study showed that lowering serum uric acid might be helpful for the prevention of calcium oxalate stones and the dissolution of radiolucent stones. However, the mechanism and the role of lowering urinary uric acid in the reduction of stone recurrence rates in patients with hypercalciuria or hyperuricosuria have not been established [214].

Urinary risk factors of kidney stone formation have been shown to associate with adherence to specific diets. The Dietary Approaches to Stop Hypertension diet reduces blood pressure and is a version of the Mediterranean diet with less animal protein and more fruits and vegetables and dairy. The relation of DASH the diet to urinary factors in men was assessed, and high adherence to the DASH dietary patterns was significantly associated with lower odds of hypocitraturia, hypercalciuria, and hyperuricosuria. In an observational, not interventional study, a more “DASH-like” dietary pattern was associated with fewer stones [175]. Moderate adherence to the DASH score increased the odds of hyperoxaluria; presumably, higher urine volume and citrate excretion negated this effect [175,215,216].

## 15. Conclusions

Twenty years down the road from the first alarming publication, kidney stone disease is becoming more prevalent worldwide, causing an increasing challenge for both patients and healthcare systems. Uncertainty remains regarding the factors responsible for this increase. Changes in dietary practices and global climate might contribute to the rise of KS and should be considered as a focus for disease prevention.

## Data Availability

Not applicable.
